# Novel prolyl endopeptidase inhibitor from *Myricaria germanica* alleviates steatohepatitis

**DOI:** 10.1039/d5ra03146j

**Published:** 2025-10-07

**Authors:** Khair Ullah, Tasneef Azam, Abdul Sammad, Tanveer Ahmed, Abdul Wadood, Muhammad Fawad Ali, Ghulam M. Mushraf, Bina Shaheen Siddiqui, Yin-xiong Li

**Affiliations:** a Center for Cell Lineage Technology and Engineering, Guangdong Provincial Key Laboratory of Biocomputing, Guangzhou Institutes of Biomedicine and Health, Chinese Academy of Sciences Guangzhou 510530 China li_yinxiong_iph@gibh.ac.cn; b University of Chinese Academy of Sciences Beijing 100049 China; c H.E.J. Research Institute of Chemistry, International Center for Chemical and Biological Sciences, University of Karachi Karachi 75270 Pakistan siddiqui_bina@yahoo.com; d Department of General Practice, The Fifth Affiliated Hospital of Southern Medical University Guangzhou 510900 China; e Department of Biochemistry, Abdul Wali Khan University Mardan Mardan-23200 Pakistan; f Cadson College of Pharmacy, University of the Punjab Lahore Pakistan; g China-New Zealand Joint Laboratory on Biomedicine and Health, State Key Laboratory of Respiratory Disease Guangzhou 510530 China

## Abstract

Prolyl endopeptidase (PREP), a serine protease, plays a critical role in the progression of hepatic steatosis and thereby contributes to metabolic dysfunction-associated fatty liver disease (MAFLD). Its inhibition has been shown to reverse disease progression. This study aimed to identify effective PREP inhibitors derived from *Myricaria germanica*, a deciduous shrub widely used in folk and traditional Chinese medicine, and to assess their potential therapeutic role in steatohepatitis. A bioassay-guided approach was employed to isolate PREP inhibitors from *M. germanica* crude extracts. The most active inhibitor was assessed through kinetic and computational studies. Moreover, its protective effects were evaluated using palmitic acid (PA) induced lipotoxicity in HepG2 cells and a high-fat diet (HFD)-induced steatohepatitis mice model. We identified and isolated a novel PREP inhibitor, (±)-2-pentacosylcyclohexanol (PREPi), with an IC_50_ value of 20.05 ± 1.6 μM. Kinetic and computational studies confirmed that PREPi acts as a competitive inhibitor. Furthermore, PREPi protected against PA-induced lipotoxicity and oxidative stress in HepG2 cells. In HFD-induced steatohepatitis mice, PREPi administration revealed improved liver function conditions (ALT, AST and ALP), quantitative scoring of steatosis and inflammation, and serum lipid profile, as well as the efficacy in weight gain and glucose tolerance. Mechanistically, PREP inhibition disrupts cascades linked to lipid accumulation and oxidative damage, suppresses lipogenic genes (SREBP-1c/FASN), and enhanced antioxidant defences positioning a novel natural PREPi as a potential candidate for steatosis and steatohepatitis treatment. These results also validate *M. germanica* as a bioactive source for intervening metabolic disorder and MAFLD.

## Abstract 2

PREPi ((±)-2-pentacosylcyclohexanol) from *Myricaria germanica* inhibits prolyl endopeptidase, alleviating steatohepatitis *via* reduced oxidative stress and lipid accumulation in preclinical models.

## Graphical abstract

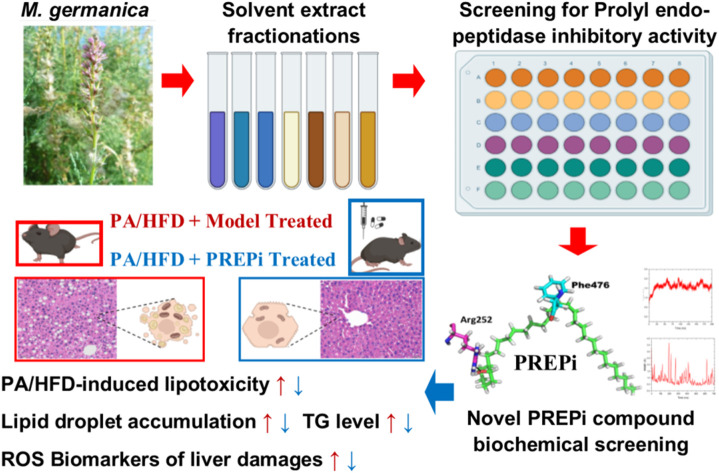

## 1 Introduction

Liver diseases, particularly metabolic-associated fatty liver disease (MAFLD) and its more severe form, non-alcoholic steatohepatitis (NASH), are characterized by hepatic steatosis and inflammation.^1,2^ Liver diseases have become a growing global health concern, with prevalence rates reaching nearly 38% in some populations. According to the latest WHO data, liver diseases are responsible for two million deaths annually and account for 4% of all deaths.^3,4^ MAFLD is closely linked to obesity, insulin resistance and metabolic disorders, progressing from simple steatosis to inflammation and fibrosis, cirrhosis and even hepatocellular carcinoma.^5–8^ Lipid accumulation, chronic inflammation, and oxidative stress-induced DNA damage are believed to be key drivers of disease progression through complex, interconnected pathways.^9–12^ Early interventions are critical to halt progression, as advanced stages often lead to irreversible liver damage and high mortality rates.^13^ However, preventing MAFLD is paramount, with dietary intervention playing a crucial role. A recent consensus reached by international experts recommends a balanced, energy-controlled diet, minimizing intake of red and processed meats, saturated and trans fats, ultra-processed foods, and alcohol.^14^

The absence of clearly defined therapeutic targets and the intricate regulatory dysfunctions involved in MAFLD have posed challenges in developing effective treatments.^8,15^ Consequently, natural products with multi-target and multi-component interactions are being explored as potential supplemental and therapeutic alternatives for MAFLD and NASH.^16–18^*M. germanica*, a wind-dispelling shrub, has been traditionally used in herbal medicine to treat various conditions, including allergic rhinitis, pain, infections or inflammatory disorders, by “removing wind” from the body.^19,20^ Recent pharmacological studies have demonstrated that *M. germanica* exhibits antibacterial, antioxidant, anti-inflammatory, and analgesic properties, particularly in models of arthritis. Additionally, leaf extracts of *M. germanica* have been utilized for treating blood anaemia, promoting liver detoxification, and managing liver-related diseases, owing to their stable quality and therapeutic efficacy. Recent research has also identified various bioactive compounds in its leaves, highlighting their potential therapeutic applications in metabolic, neurodegenerative, and other diseases.^20^

Prolyl endopeptidase (PREP; EC 3.4.21.26), a serine protease, selectively hydrolyzes peptide bonds at the carboxyl terminus of proline residues in polypeptides, including key bioactive molecules such as substance P, neuropeptides, and regulatory proteins.^21,22^ Although PREP has been extensively studied in the context of neurodegenerative diseases, emerging evidence highlights its significant role in liver function and pathology.^23,24^ PREP is highly expressed in hepatic tissues, where it plays a crucial role in cellular homeostasis, metabolic regulation, and inflammatory responses.^25,26^ In MAFLD, chronic lipid accumulation leads to hepatocyte dysfunction, oxidative stress, and inflammatory cascades that progressively damage liver tissue.^10,27^ PREP is implicated in these processes through multiple mechanisms. First, it modulates oxidative stress by influencing redox homeostasis and reactive oxygen species (ROS) production. Studies indicate that PREP inhibition can mitigate ROS generation, thereby reducing oxidative damage, a key driver of fatty liver progression. Second, PREP is involved in regulating autophagy, a cellular degradation process critical for clearing damaged organelles and maintaining metabolic balance.^23,28^ Dysregulated autophagy contributes to hepatocyte lipid accumulation and inflammation, exacerbating MAFLD pathology and progression towards irreversible NASH.^29,30^ PREP plays a role in hepatic lipid metabolism, where it protects against high-fat diet (HFD) induced lipid dysregulation and metabolic disorders. PREP's contribution to lipid accumulation is linked to its influence on key signalling pathways that regulate fatty acid oxidation, lipogenesis, and lipid storage. Disruption of PREP activity has been shown to prevent hepatocyte steatosis induced by oleic acid (OA) and palmitic acid (PA) *in vitro*, suggesting that targeting PREP may provide therapeutic benefits in MAFLD.^28,31^ Additionally, PREP is associated with inflammatory signalling pathways that exacerbate liver fibrosis and disease progression. Chronic inflammation is a hallmark of NASH, driven by immune cell infiltration and the persistent activation of pro-inflammatory cytokines. PREP influences these inflammatory pathways, and its inhibition has been found to reduce hepatic inflammation and fibrosis, providing a potential strategy for slowing disease progression.^26^ Another critical aspect of PREP's role in steatohepatitis and NASH involves its interaction with the tumour suppressor protein P53. P53 is a key regulator of oxidative stress and inflammation, activating downstream antioxidant signalling pathways such as Sesn2/Nrf2/HO-1.^32–34^ Interestingly, PREP inhibition has been shown to upregulate P53 expression, suggesting that PREP modulates oxidative stress through P53 and its associated protective pathways.^26^ This link further highlights PREP as a potential therapeutic target for reducing oxidative damage and inflammation in MAFLD and NASH, leading to hepatic cirrhosis.

While synthetic PREP inhibitors have shown promise in mitigating steatohepatitis-related liver damage,^28^ however, there is growing interest in identifying natural PREP inhibitors that offer improved safety and efficacy. Natural compounds with PREP-inhibitory activity could provide a novel, multi-target approach to addressing the complex pathophysiology of NASH. In this study, a bioassay-guided approach was employed to isolate and identify an effective PREP inhibitors from *M. germanica* and evaluate their therapeutic effects on steatosis and steatohepatitis. A novel PREP inhibitor, (±)-2-pentacosylcyclohexanol (PREPi), with an IC_50_ value of 20.05 ± 1.6 μM. Kinetic and computational studies confirmed that PREPi acts as a competitive inhibitor. Furthermore, PREPi protected against PA-induced lipotoxicity and oxidative stress in HepG2 cells. In HFD-induced steatohepatitis mice, PREPi administration revealed improved liver function conditions (ALT, AST and ALP), quantitative scoring of steatosis and inflammation, and serum lipid profile, as well as the efficacy in weight gain and glucose tolerance. Mechanistically, PREP inhibition disrupts cascades linked to lipid accumulation and oxidative damage, suppresses lipogenic genes (SREBP-1c/FASN), and enhanced antioxidant defences positioning a novel natural PREPi as a potential candidate for steatosis and steatohepatitis treatment.

## 2. Materials and methods

### 2.1. Materials

The solvents dichloromethane, methanol, n-butanol, petroleum ether, and ethyl acetate were obtained from commercial sources. Thin-layer chromatography (TLC) was performed using pre-coated silica gel aluminium sheets from E. Merck (0.5 mm thickness, 20 × 20 cm), and silica gel column chromatography (mesh sizes 70–230 and 240–300) was employed for separation, also from E. Merck. Recombinant human prolyl endopeptidase, Z-Gly-Pro-pNA (a substrate of PREP), Kyp-2047 (standard inhibitor), MTT [3-(4,5-dimethylthiazol-2-yl)-2,5-diphenyl tetrazolium bromide], cycloheximide, and Nile red were obtained from Sigma-Aldrich, USA. HepG2 cell lines were kindly provided by Dr Pan from the Guangzhou Institutes of Biomedicine and Health, Chinese Academy of Sciences, Guangzhou. Low-glucose Dulbecco's Modified Eagle's Medium (DMEM-L) and fetal bovine serum (FBS) were procured from Grand Island, NY (Catalogue number: 16000044).

### 2.2. Collection of plant material and activity-guided isolation of PREP inhibitors

The leaves of *M. germanica* (L.) Desv. (6.0 kg) were collected in mid-May 2016 from the Bulchi Bagrote Valley in Gilgit-Baltistan, Pakistan. The plant material was identified by Sher Wali Khan, Head of the Department of Environmental Sciences at Karakoram International University (KIU), Gilgit-Baltistan. A voucher specimen (No. 22) was deposited in the KIU herbarium for future reference. The collected leaves (6.0 kg) were air-dried at room temperature (∼25 °C), yielding 5.5 kg of dried material. These dried leaves (5.0 kg) were extracted with methanol through three successive 10 days soaking periods. After solvent evaporation under reduced pressure, the resulting crude extract (600 g) was partitioned between ethyl acetate and water (1 : 3, v/v). The ethyl acetate (EtOAc) fraction, which showed higher inhibitory activity, was adsorbed onto Na_2_CO_3_ and eluted with a chloroform-methanol gradient. The resulting filtrate was further partitioned into petroleum ether-soluble and insoluble fractions. These fractions were subjected to normal-phase column chromatography using two solvent systems: petroleum ether-EtOAc gradient: starting from 100% petroleum ether to 9 : 1 petroleum ether-EtOAc. While DCM: MeOH gradient: starting from 100 : 0 to 1 : 100 (v/v). Furthermore, fractions were analyzed by thin-layer chromatography (TLC) and tested for PREP inhibitory activity at concentrations of 500 μg mL^−1^.

### 2.3. Characterization techniques

The 1H-NMR and 13C-NMR spectra of the compounds were recorded on Bruker AM-400 and AM-500 spectrometers at 500 MHz. IR spectra were obtained using a JASCO 302 A spectrometer with KBr discs. Mass spectra were acquired on a Finnigan MAT 311 and JEOL JMS 600H-1 spectrometer. GC analysis was performed using a Shimadzu GC-10 system with an FID detector, and separations were done on a 60 m OPTIMA®-5-Accent capillary column. GC-MS was carried out with an Agilent 6890 GC coupled to a JMS-600H mass spectrometer, using a ZB-5MS column. The mass spectrometer operated in EI mode with an ion source temperature of 250 °C and electron energy of 70 eV, and the carrier gas flow rate was between 1.0 and 5.0 mL min^−1^.

### 2.4. *In vitro* PREP inhibition assay and kinetic studies

The PREP inhibition assay was performed in a 96-well plate with slight modifications to the enzyme and substrate concentrations.^35^ In a 200 μL reaction mixture, sodium phosphate buffer (pH 7.0) containing PREP and the sample solution was incubated at 30 °C for 10 minutes. After recording the absorbance, a substrate solution was added. Optical density (O.D.) changes were recorded at 410 nm for 30 minutes using a Multiskan GO UV/Vis Spectrophotometer. The percentage inhibition was calculated using the formula:% Inhibition = 100 × (O.D. of test compound/O.D. of control) − 100

For kinetic studies, the PREP enzyme was incubated with different concentrations of the test compound at 30 °C for 10 minutes. The reaction was initiated by adding the substrate. Catalytic activity was measured at 410 nm using the Multiskan GO UV/Vis Spectrophotometer.

### 2.5. Computational studies

#### 2.5.1 Enzyme structure preparation and minimization

The 3D structure of PREP (PDB ID: 3DDU, resolution 1.56 Å was downloaded from the Protein Data Bank (https://www.rcsb.org/).^36^ The crystal structure has been found with missing residues at positions 425 and 428, which were modelled using Chimera. The structure was refined using Amber14: EHT (Amber ff14SB and ETH combined) protein force field Autodock.^37,38^ The enzyme was minimized by adding the missing hydrogen.

#### 2.5.2 Molecular docking

The molecular docking studies were performed to find out the preferred binding interaction of the nominated ligand, *i.e.* PREPi, with the active site residue of the targeted enzyme. Ligand-based rigid docking was performed using the default docking protocol implemented in Autodock. It is of great importance to validate the docking protocol. Here, we found our docking protocol valid by re-docking the co-crystalized ligand in the binding pocket of the protein. Using Pymol, the RMSD between the co-crystal and re-docked conformation was calculated as 0.593 Å, suggesting that our docking protocol is reliable. Ensuring docking reliability docking PREPi was docked into the active site of PREP using the default docking protocol of Autodock. A total of 10 conformations were saved for the assessing ligand.

#### 2.5.3 Molecular dynamics simulations

To better understand the dynamics behaviour and energetics of ligand–enzyme complex all atom's molecular dynamics were performed MD simulations and related analyses for the systems were performed using AMBER v2022 with the force field (ff14SB).^39^ For neutralization of the system, counter ions (Na^+^ and Cl^−^) were added using tLEAP. For the solvation of the system, the octahedral box of the TIP3P water model with a 12.0 Å buffer was used. A cutoff distance of 10 Å was used for the van der Waals and long-range electrostatic interactions. The Particle Mesh Ewald PME algorithm was used for the treatment of long-range electrostatic interactions.^40^ To constrain the bonds involving hydrogen atoms SHAKE algorithm was used, followed by a constant pressure of 2 ns equilibrated at 300 K.^41^ Langevin dynamics was used for controlling the temperature.^42^ Finally, 200 ns MD simulation was run using CUDA version of PMEMD for the equilibrated complex at constant temperature and pressure.^43^ MD trajectories were analyzed using the CPPTRAJ module of Amber v2022. The analyses that were performed included RMSD, RMSF, RoG and DCCM.

### 2.6. Cell culture and steatohepatitis model development

The HepG2 cells were cultured in Dulbecco's Modified Eagle Medium (DMEM) supplemented with 10% fetal bovine serum (Thermo Fisher Scientific, Waltham, MA, USA) and 1% penicillin-streptomycin (Biological Industries, Kibbutz Beit Haemek, Israel). The cells were maintained at 37 °C in a humidified atmosphere containing 5% CO_2_.

To establish the steatohepatitis model, palmitic acid (PA; Cayman Chemical Company, Ann Arbor, MI, USA) was conjugated with 1% bovine serum albumin (BSA; US Biological Life Sciences, Swampscott, MA, USA) in complete DMEM medium before treatment. HepG2 cells were used between passages 5 and 10 for all experiments involving PA-induced steatohepatitis conditions.

Cells were then exposed to BSA-conjugated PA (500 μM) for 24 hours to induce lipotoxicity and oxidative stress, mimicking the *in vitro* conditions of steatohepatitis. The effect of the PREP inhibitor (PREPi) was evaluated in combination with PA. Control cells were treated with 1% BSA alone.

### 2.7. Cell cytotoxicity detection

HepG2 cells were seeded at a density of 1 × 10^4^ cells per well in a 96-well plate and incubated overnight at 37 °C in a 5% CO_2_ atmosphere. The cells were subsequently treated with 500 μM PA alone or in combination with 15 μM PREPi for 24 hours. After the treatment period, 10 μL of CCK-8 solution (Dojindo, Japan) was added to each well, and the plate was incubated for an additional hour at 37 °C. Absorbance was measured at 450 nm using a microplate reader, and cell viability was calculated relative to untreated controls. All treatments were performed in triplicate.

### 2.8. Detection of reactive oxygen species (ROS) assay

Intracellular ROS levels in cells treated with PA and PREPi were measured using the fluorescent probe 2′,7′-dichlorodihydrofluorescein diacetate (DCFH-DA). Cells were incubated with 10 μM DCFH-DA for 30 minutes at 37 °C in the dark. Following incubation, the cells were washed twice with phosphate-buffered saline (PBS) to remove any excess probe. Fluorescence images were acquired using a fluorescence microscope at a magnification of ×200, and the fluorescence intensity was quantified using ImageJ software. The results were normalized to the control and expressed as a percentage.

### 2.9. Nile red staining

After lipid induction, cells were washed with PBS, fixed with 4% paraformaldehyde, and incubated with a 1 μg mL^−1^ Nile red solution for 30 minutes in the dark. The nuclei were counterstained with DAPI, and cells were washed again with PBS. Stained cells were imaged using a fluorescence microscope (excitation: 488 nm, emission: 560 nm), and lipid droplets were visualized as bright red fluorescent structures. ImageJ software was used to quantify lipid droplets. Additionally, Nile red-stained cells were analyzed by fluorescence-activated cell sorting (FACS) to assess lipid accumulation and cell population characteristics.

### 2.10. Intracellular triglyceride (TG) quantification

TG levels in HepG2 cells were measured using a TG assay kit (Asanpharm, Gyeonggi-do, Korea). Briefly, after 24 hours of treatment with PA alone or in combination with PREPi, the cells were washed twice with PBS and harvested using a cell scraper. TG extraction was performed by adding a chloroform-methanol mixture (2 : 1), followed by vortexing and centrifugation at 2000×*g* for 10 minutes. The organic phase was collected and mixed with the assay kit reagents according to the manufacturer's instructions. The mixture was incubated at 37 °C for 10 minutes, and absorbance was measured at 540 nm. TG concentrations were determined using a standard curve.

### 2.11. RNA extraction and real-time quantitative PCR

Total RNA was extracted from HepG2 cells using the Trizol reagent (Invitrogen, USA) according to the manufacturer's instructions. The extracted RNA was then reverse-transcribed into cDNA, which was subsequently amplified by PCR. Real-time PCR was performed using a Bio-Rad CFX96 system. The relative mRNA expression levels of target genes were analyzed by normalizing to the housekeeping gene GAPDH. Details of the primers used in this study are provided in Table 1.

**Table 1:** Primers used for quantitative real-time PCR. Forward and reverse sequences are listed in the 5′ to 3′ direction

Gene name	Forward sequence (5′–3′)	Reverse sequence (5′–3′)
PREP	GACCACTGATTACGGGTGCT	AGAAGCATGGACGGGTACTG
SREBP-1c	GCGCCTTGACAGGTGAAGTC	GCCAGGGAAGTCACTGTCTTG
FASN	CACCCTGATTTCTGCCATCT	AATGTGCTTGGCTTGGTAGC
TNF-α	TGTCCCTTTCACTCACTGGC	CATCTTTTGGGGGAGTGCCT
NFKB1	ATTTGAAACACTGGAAGCACGG	CCGCCTTCTGCTTGTAGATAGG
IL-6	ACTCACCTCTTCAGAACGAATTG	CCATCTTTGGAAGGTTCAGGTTG
Nrf2	CACATCCAGTCAGAAACCAGTGG	GGAATGTCTGCGCCAAAAGCTG

### 2.12. Animals and experimental design

A murine model using male C57BL/6 mice was established to study steatohepatitis and evaluate PREPi treatments. The animals were housed under controlled environmental conditions, with a temperature of 25 ± 0.5 °C, humidity of 55 ± 5%, and a 12 h : 12 h light–dark cycle. Mice were randomly assigned to three experimental groups (*n* = 6 per group): control, model, and PREPi treatment groups. The model and PREPi treatment groups were initially fed a high-fat diet (HFD) for 4 weeks to induce MAFLD-like features, including hepatic steatosis, inflammation, and metabolic dysregulation. Following this initial period, the PREPi treatment group received intravenous (IV) injections of 6.757 mg kg^−1^ PREPi for an additional 20 weeks, while continuing on the HFD. The model group continued on the HFD for a total of 24 weeks without PREPi treatment. The control group was maintained on a standard chow diet throughout the 24 week experimental period. To account for the effects of the drug vehicle, both the control and model groups received IV injections of 4% DMSO in saline water, matching the volume and frequency of the PREPi treatment group. Throughout the study, body weight, food intake, and metabolic parameters were monitored. After 24 weeks, all mice were euthanized, and liver tissues were harvested for histological analysis, biochemical assays, and molecular studies to assess lipid accumulation, inflammatory markers, and fibrotic changes. Mice were anaesthetized *via* intraperitoneal injection using a ketamine-xylazine cocktail at dosages of approximately 90 mg kg^−1^ of ketamine and 7 mg kg^−1^ of xylazine. Furthermore, all animal studies, including mice feeding and operation experiments, were approved by the experimental animal ethics committee of the Guangzhou Institutes of Biomedicine and Health (GIBH) under the Chinese Academy of Sciences (CAS).

### 2.13. Measurement of biochemical parameters in plasma and serum

The levels of serum biochemical parameters, such as total cholesterol (TC), triglycerides (TG), low-density lipoprotein cholesterol (LDL-C), high-density lipoprotein cholesterol (HDL-C), alanine aminotransferase (ALT), and aspartate aminotransferase (AST), were measured using an automated biochemical analyzer (Hitachi 7020, Tokyo, Japan). Furthermore, the glucose tolerance test (GTT) and pyruvate tolerance test (PTT) were performed on 16 h-fasted mice. Mice were intravenously injected with 2 g kg^−1^ body weight glucose (Sigma-Aldrich, USA) or with 6.75/kg body weight insulin (Sigma-Aldrich, USA). GTT and PTT were measured from tail blood at 0, 15, 30, 60, 90 or 120 min after the glucose or pyruvate administration. The area under the curve (AUC) was calculated to quantify the GTT and PTT results.

### 2.14. Histopathology

A consistent section of the liver lobe was collected from each animal and promptly placed in a 10% neutral buffered formaldehyde solution. The tissue samples were then fixed and embedded in paraffin. Sections with a thickness of 4 to 5 μm were cut and placed on glass slides. These slides were stained using the hematoxylin-eosin (H&E) staining technique. Images were taken at 400× magnification using an optical microscope. Histopathological scoring was performed in a blinded fashion based on the NAFLD Activity Score (NAS) system,^44^ Kleiner *et al.*, 2005), which evaluates macrovesicular steatosis (0–3), lobular inflammation (0–3), and hepatocellular ballooning (0–2), yielding a total score ranging from 0 to 8.

### 2.15. Statistical analysis

The data was labelled with mean ± standard deviation, and the experimental data was statistically analyzed using GraphPad Prime 8.0 software. GraFit 7 was used for plotting the enzyme kinetics graphs. The significance between the two groups was assessed with an unpaired student's *t*-test, and among three or more groups was assessed with one-way ANOVA. All data represent means ± SD. Statistical significance is denoted by *p < 0.05, **p < 0.01, ***p < 0.001, and ****p < 0.0001.

## 3. Results and discussion

### 3.1. Activity-guided isolation of PREP inhibitors

The ethyl acetate (EtOAc) fraction of *M. germanica* leaf extract demonstrated remarkable PREP inhibitory activity, with 97.8% inhibition at 1 mg mL^−1^ compared to the aqueous phase (42.6%). This significant activity justified its selection for further purification. Bioactivity-guided fractionation of the EtOAc extract yielded four compounds with varying degrees of PREP inhibitory activity. From the pet ether insoluble fraction (OSI salt fraction), three compounds were isolated: methyl gallate (Fr-1, 59.0% inhibition at 500 μg mL^−1^, 24.7% at 200 μM), syringic acid (Fr-2, 75.4% inhibition at 500 μg mL^−1^, IC_50_ = 155.13 ± 1.8 μM), and butanedioic acid (Fr-3, 64.7% inhibition at 500 μg mL^−1^, 34.3% at 200 μM). These compounds exhibited weak to moderate PREP inhibitory activity, consistent with previous reports on phenolic compounds as PREP inhibitors. The pet ether soluble fraction (7% salt fraction) yielded four sub-fractions, with Fr-1 showing the highest inhibitory activity (88.6% inhibition at 500 μg mL^−1^). This fraction led to the isolation of (±)-2-pentacosylcyclohexanol (PREPi), which demonstrated potent PREP inhibitory activity with an IC_50_ value of 20.05 ± 1.6 μM, making it the most promising bioactive compound isolated from *M. germanica*. PREPi's potency was approximately tenfold greater than syringic acid and significantly higher than other isolated compounds, suggesting that its unique structural features contribute to its strong binding affinity to PREP. The detailed schematic descriptions are provided in SI Fig. 1.

### 3.2. Structural elucidation of the isolated PREP inhibitors

**Fig. 1:**
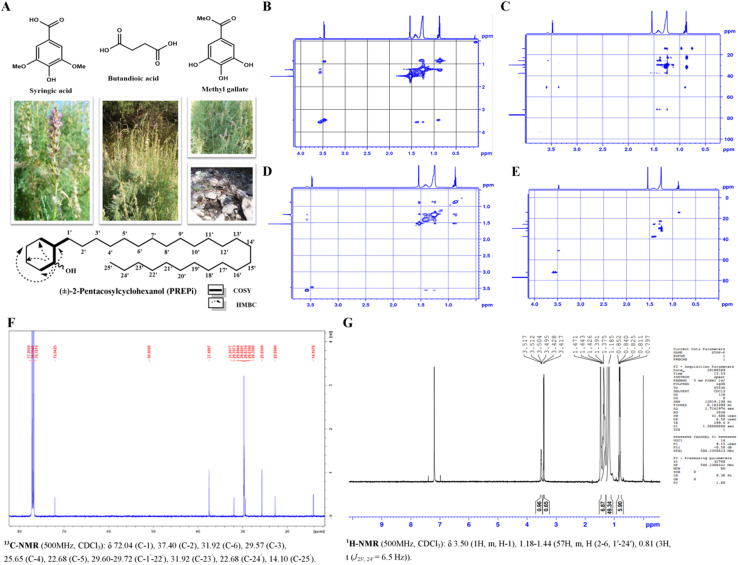
Characterization of *M germanica* and its active compound, (±)-2-pentacosylcyclohexanol (PREPi). (A) Different life stages and growth conditions of *M. germanica* and the chemical structure of PREPi. (B) Correlation Spectroscopy (COSY) determines the connectivity between hydrogen atoms in PREPi. (C) Heteronuclear Multiple Bond Correlation (HMBC) of PREPi. (D) Nuclear Overhauser Effect Spectroscopy (NOSY) spectra of PREPi. (E) Heteronuclear Single Quantum Coherence (HSQC) of PREPi. (F and G) ^13^C NMR and ^1^H NMR spectrum of PREPi.

PREPi was obtained as a white powder and identified as a new compound with the molecular formula C_31_H_62_O, as determined by high-resolution electron ionization mass spectrometry (HREI-MS). The EI-MS spectrum displayed a molecular ion peak [M^+^] at *m*/*z* 450.0. The IR spectrum exhibited a hydroxyl absorption peak at 3449.9 cm^−1^, while asymmetric and symmetric stretching vibrations for methylene groups were observed at 2918.3 cm^−1^ and 2849.8 cm^−1^, respectively. The UV spectrum (in MeOH) showed an absorption band at 260 nm, indicative of the presence of a hydroxyl group. The ^13^C NMR spectrum reveals the carbon environment, while COSY identifies proton–proton couplings, and HMBC offers insights into long-range hydrogen-carbon correlations. NOSY highlights through-space proton interactions, and HSQC establishes direct proton–carbon relationships. Together, these techniques confirm the molecular structure of PREPi, elucidating the connectivity and spatial arrangement of atoms within the compound (Fig. 1B–E).

The ^1^H-NMR spectrum displayed four distinct signals. The proton at C-1 appeared as a multiplet at *δ* 3.50 ppm (1H), while the hydroxyl group (OH) resonated as a doublet at *δ* 3.42 ppm. The protons of C-3, C-4, C-5, and C-6 collectively produced an intense singlet at *δ* 1.47 ppm, and the proton at C-2 appeared as a multiplet at *δ* 1.36 ppm (Fig. 1G and Table 2). In the ^13^C-NMR spectrum, the signals for C-1 and C-2 were observed at *δ* 72.0 ppm and *δ* 50.90 ppm, respectively. The signal for C-6 appeared at *δ* 37.48 ppm, while C-1′ resonated at *δ* 31.91 ppm. The signals for C-2′ to C-24′ were observed at *δ* 29.67 ppm. The signals for C-4, C-3, and C-5 appeared at *δ* 25.65 ppm, *δ* 22.75 ppm, and *δ* 22.68 ppm, respectively, and the signal for C-25′ was observed at *δ* 14.10 ppm (Fig. 1G and Table 2). The C-13 (Fig. 1F and Table 2), ^1^H-NMR Correlation Spectroscopy (COSY) spectra (Fig. 1B), Heteronuclear Multiple Bond Correlation (HMBC) (Fig. 1C), Nuclear Overhauser Effect Spectroscopy (NOSY) spectra (Fig. 1D), Heteronuclear Single Quantum Coherence (HSQC) spectra of (Fig. 1E) of the PREPi are due provided.

**Table 2:** ^1^H NMR and ^13^C NMR spectroscopic data of PREPi, methyl gallate, syringic acid, and butanedioic acid. *δ*_H_: Proton (^1^H) NMR chemical shift. *δ*_C_: Carbon (^13^C) NMR chemical shift. s, d, m: Singlet, doublet, multiplet, respectively. Numbers in parentheses represent the number of protons

Position	PREPi	Methyl gallate	Syringic acid	Butanedioic acid
1	*δ*_H_: 119.3, *δ*_C_: 120.6, *δ*_H_: 173.9, *δ*_C_: 3.50, *δ*_H_: 72.0	*δ*_H_: 7.02, *δ*_C_: 108.5	*δ*_H_: 7.15, *δ*_C_: 106.9	*δ*_H_: 7.02, *δ*_C_: 108.5
2	*δ*_H_: (1H, m)	*δ*_H_: (1H, s)	*δ*_H_: (1H, d, 0.6)	*δ*_H_: (1H, m)
3	*δ*_H_: 145.7, *δ*_C_: 147.3, *δ*_H_: 2.55 (2H, s), *δ*_C_: 28.9	*δ*_H_: 145.7, *δ*_C_: 147.3	*δ*_H_: 145.7, *δ*_C_: 137.4	*δ*_H_: 137.4, *δ*_C_: 137.5
4	*δ*_H_: 1.47, *δ*_C_: 22.75	*δ*_H_: 2.55 (2H, s), *δ*_C_: 28.9	*δ*_H_: 1.47, *δ*_C_: 22.75	*δ*_H_: 1.47, *δ*_C_: 25.65
5	*δ*_H_: 22.75, *δ*_C_: 22.68	*δ*_H_: 1.47, *δ*_C_: 22.68	*δ*_H_: 1.47, *δ*_C_: 22.68	*δ*_H_: 1.47, *δ*_C_: 22.68
6	*δ*_H_: (1H, m)	*δ*_H_: 7.02, *δ*_C_: 108.5	*δ*_H_: (1H, d, 0.6)	*δ*_H_: (1H, m)
7	*δ*_H_: 7.02, *δ*_C_: 108.5	*δ*_H_: 7.15, *δ*_C_: 106.9	*δ*_H_: 7.15, *δ*_C_: 106.9	*δ*_H_: 7.15, *δ*_C_: 106.9
8	*δ*_H_: 166.4, *δ*_C_: 167.9	*δ*_H_: 166.4, *δ*_C_: 167.9	*δ*_H_: 166.4, *δ*_C_: 167.9	*δ*_H_: 166.4, *δ*_C_: 167.9
9	*δ*_H_: 3.80, *δ*_C_: 52.3	*δ*_H_: 3.80, *δ*_C_: 52.3	*δ*_H_: 3.86, *δ*_C_: 56.2	*δ*_H_: 3.86, *δ*_C_: 56.2
10	*δ*_H_: (3H, s), *δ*_C_: 56.2	*δ*_H_: (3H, s), *δ*_C_: 56.2	*δ*_H_: (3H, s), *δ*_C_: 56.2	*δ*_H_: (3H, s), *δ*_C_: 56.2
11	*δ*_H_: 3.83, *δ*_C_: 56.2	*δ*_H_: 3.83, *δ*_C_: 56.2	*δ*_H_: 3.83, *δ*_C_: 56.2	*δ*_H_: 3.83, *δ*_C_: 56.2
12	*δ*_H_: 56.2	*δ*_H_: 56.2	*δ*_H_: 56.2	*δ*_H_: 56.2
13	*δ*_H_: 1.18, *δ*_C_: 29.67	*δ*_H_: 1.18, *δ*_C_: 29.67	*δ*_H_: 1.18, *δ*_C_: 29.67	*δ*_H_: 1.18, *δ*_C_: 29.67
14	*δ*_H_: 29.67	*δ*_H_: 29.67	*δ*_H_: 29.67	*δ*_H_: 29.67
15	*δ*_H_: 0.81, *δ*_C_: 14.10	*δ*_H_: 0.81, *δ*_C_: 14.10	*δ*_H_: 0.81, *δ*_C_: 14.10	*δ*_H_: 0.81, *δ*_C_: 14.10
16	*δ*_H_: 14.10	*δ*_H_: 14.10	*δ*_H_: 14.10	*δ*_H_: 14.10

Methyl gallate was isolated as a white solid with the molecular formula C_8_H_8_O_5_, as determined by high-resolution electron ionization mass spectrometry (HREI-MS). The EI-MS spectrum showed a molecular ion peak [M^+^] at *m*/*z* 184. The IR spectrum exhibited characteristic absorption peaks at 3400 cm^−1^ (O–H stretching), 1710.6 cm^−1^ (C=O stretching), 3055.5 cm^−1^ (aromatic C–H stretching), and 2870 cm^−1^ (asymmetric C–H stretching). In the 1H-NMR spectrum, a singlet at *δ* 7.02 ppm corresponded to the aromatic ring protons, while the methyl group protons appeared as a singlet at *δ* 3.80 ppm. In the 13C-NMR spectrum, the signal for C-7 was observed at *δ* 169.0 ppm, while C-3 and C-5 resonated at *δ* 146.47 ppm. The signal for C-4 appeared at *δ* 139.74 ppm, and C-1 was observed at *δ* 121.44 ppm. The signals for C-2 and C-6 appeared at *δ* 110.03 ppm, and the signal for C-8 was observed at *δ* 52.24 ppm. Syringic acid was isolated as a white solid. The EI-MS spectrum displayed a molecular ion peak [M^+^] at *m*/*z* 198.0, and the molecular formula was determined to be C_9_H_10_O_5_ using high-resolution electron ionization mass spectrometry (HREI-MS). The IR spectrum exhibited characteristic absorption peaks at 3400 cm^−1^ (O–H stretching), 1715.6 cm^−1^ (C=O stretching), 3058.5 cm^−1^ (aromatic C–H stretching), and 2860 cm^−1^ (asymmetric C–H stretching). In the 1H-NMR spectrum, a singlet at *δ* 7.15 ppm corresponded to the aromatic ring protons, while the protons of the two methyl groups appeared as a singlet at *δ* 3.84 ppm. In the ^13^C-NMR spectrum, the signal for C-7 was observed at *δ* 168.84 ppm, while C-3 and C-5 resonated at *δ* 149.12 ppm. The signal for C-4 appeared at *δ* 140.61 ppm, and C-1 was observed at *δ* 120.6 ppm. The signals for C-2 and C-6 appeared at *δ* 106.08 ppm, and the signals for C-8 and C-9 were observed at *δ* 56.66 ppm. Butanedioic acid was purified as a white powder with the molecular formula C_4_H_6_O_4_, as determined by HREI-MS. The EI-MS spectrum showed a molecular ion peak [M^+^] at *m*/*z* 100.0. The IR spectrum exhibited absorption peaks at 3400 cm^−1^ (O–H stretching), 1710 cm^−1^ (C=O stretching), and 2850 cm^−1^ (asymmetric C–H stretching). In the ^1^H-NMR spectrum, a triplet at *δ* 2.55 ppm corresponded to the protons at C-2 and C-3. In the 13C-NMR spectrum, the signals for C-1 and C-4 appeared at *δ* 173.9 ppm, while the signals for C-2 and C-3 were observed at *δ* 28.9 ppm (Table 2).

### 3.3. *In Vitro* PREP inhibition and kinetic results

**Fig. 2:**
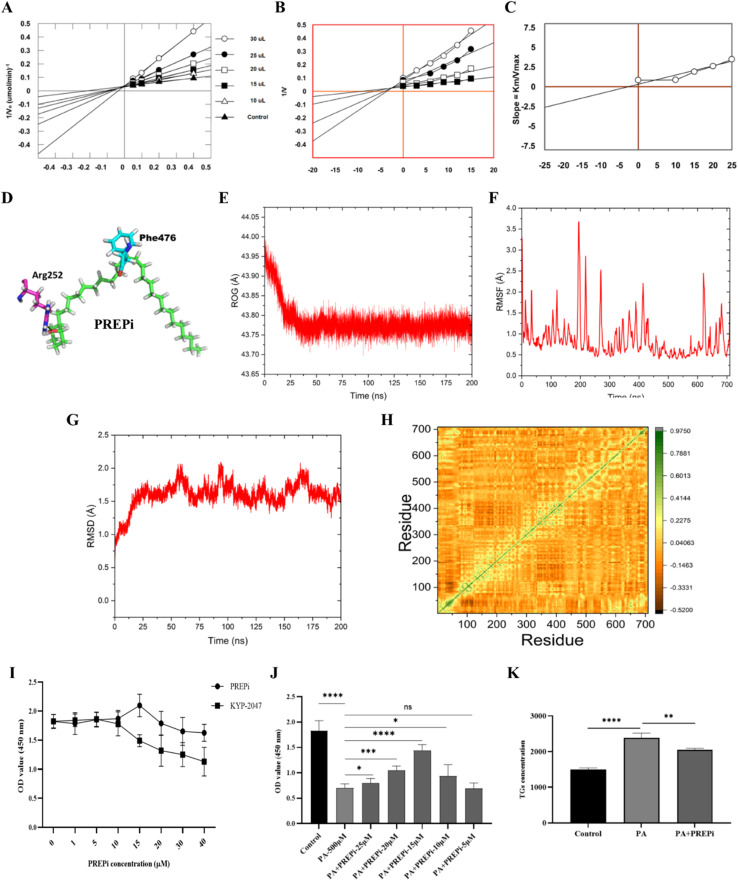
Molecular docking, enzyme inhibition, and cell-based studies of PREPi. (A) Lineweaver–Burk plot of PREPi inhibition at various PREPi concentrations. (B) Detailed Lineweaver–Burk plot of PREPi inhibition focusing on the concentration range, highlighting the shift in slope at different inhibitor concentrations. (C) Secondary plot showing the slope of the Lineweaver–Burk plot *versus* the PREPi concentration. (D) Predicted binding interactions of (±)-2-pentacosylcyclohexanol (PREPi) within the active site of prolyl endopeptidase (PREP), showing key hydrogen bonds and hydrophobic interactions. (E) Radius of Gyration (RoG) plot illustrating the compactness of the PREPi–PREP complex. (2F) RMSF (Root Mean Square Fluctuation) analysis showing minimal flexibility at the active site of PREP upon PREPi binding. (G) RMSD (Root Mean Square Deviation) analysis indicating the stability of the PREPi-PREP complex during molecular dynamics simulations (H) Dynamic Cross-Correlation Matrix (DCCM) analysis between active site residues upon PREPi binding. (I) HEPG2 cell viability at various concentrations of PREPi and Kyp-2047. (J) Effect of varying doses of PREPi on PA-induced lipotoxicity model in HepG2 cells. (K) Triglyceride (TG) quantification in PA-induced HepG2 cells. All data are presented as means ± SD. Statistical significance is indicated as follows: *p* < 0.05 (*), *p* < 0.01 (**), *p* < 0.001 (***), *p* < 0.0001 (****), “ns” denotes non-significant difference.

The *in vitro* PREP inhibition activity of four compounds isolated from *M germanica* was evaluated. The compound (±)-2-pentacosylcyclohexanol (PREPi) exhibited the highest percent inhibition at 92.0%, with an IC_50_ value of 20.05 ± 1.6 μM. The other three compounds (methyl gallate, syringic acid, and butanedioic acid) were found inactive against PREP inhibitory activities with percent inhibition of 28.6%, 66.5% (IC_50_ value of 155.13 ± 1.8 μM), and 43.3%, respectively. These findings highlight that PREPi is the most potent inhibitor of PREP among all the compounds tested. The competitive inhibitory mechanism of PREPi was confirmed through detailed kinetic analysis. Lineweaver–Burk plots confirmed the competitive nature of inhibition, showing increased *K*_m_ values without affecting *V*_max_ (Fig. 2A–C). This data suggested that PREPi directly interferes with substrate binding, further validating its potential as a targeted PREP inhibitor.

### 3.4. Computational studies

To further validate the *in vitro* kinetic experiments, molecular docking was performed. The compound PREPi was docked into the active site of PREP. PREPi is a long-chain alcohol having a hydroxyl group attached to the aromatic ring. During docking interactions, it has been found that the hydroxyl group attached to the aromatic ring forms a strong hydrogen bond with the nitrogen of active site residue *i.e.* Arg252 releasing −0.7 kcal mol^−1^ energy, which indicates a stronger interaction, along with carbon-18 of alpha carbon chain forms a hydrophobic interaction with the pi electrons of aromatic ring of Phe 476 with the release of −0.5 kcal mol^−1^ energy suggesting a strong and stable interaction of PREPi with the active site residues of the enzyme. Along with the compound has been found to have a minimum docking score of −8.827 which further guarantees its strong interaction with the enzyme. Protein–ligand interaction has been shown in Fig. 2D.

Molecular dynamics (MD) simulations were conducted to investigate the dynamic behaviour and energetics of a ligand–enzyme complex. The simulations were performed using AMBER v2022, with the force field ff14SB. The aim was to assess the stability, flexibility, and binding characteristics of the ligand–enzyme complex over a 200 ns simulation period. The following necessary analyses were performed.

The Radius of Gyration (RoG) measures the compactness and flexibility of the protein, with lower values indicating a more rigid structure. The RoG results demonstrate that the ligand successfully folded and bound to the predicted binding site, leading to increased compactness of the 3DDU (prolyl endopeptidase) and the PREPi complex. This indicates a stable and well-bound complex throughout the simulation as shown in Fig. 2E.

Root Mean Square Fluctuation (RMSF) quantifies the flexibility of individual residues or regions within the protein structure. The analysis revealed that the compound PREPi exhibited consistently low RMSF values throughout the simulation trajectory, indicating overall structural stability. The highest fluctuations were observed at residues 207 and 280, with a maximum deviation of approximately 2.5 Å. However, these fluctuations were of minimal significance as they did not involve active site residues and thus did not compromise the overall stability of the protein. Importantly, no hyper-fluctuations were detected, particularly at the active site residues, reinforcing the structural integrity of the complex. These findings suggest that the binding of PREPi maintains a highly stable protein conformation with minimal perturbations, further supporting its potential as a stable ligand. The RMSF graph illustrating these fluctuations is presented in Fig. 2F.

RMSD measures the deviation of the structure from a reference, indicating the stability of the protein relative to its conformation over time. The RMSD graph indicated that the ligand–enzyme complex was stable from 0 ns to 40 ns, with an RMSD change of 1.2 Å. Minor fluctuations were observed from 40 ns to 45 ns, with an RMSD exceeding 1.5 Å. RMSD deviation and stabilization were observed throughout 170 ns, and after that, the complex remained stable until the end of the simulation, as shown in Fig. 2G. Due to the strong interaction of PREPi, specifically of alpha C-18 with the aromatic ring of Phe_476_, it greatly reduces the deviation and hence results in stable RMSD throughout the trajectories with fewer exceptions as discussed formerly.

The DCCM analyzes the correlation between movements of residues in the binding pocket of the target protein. The DCCM revealed regions of positively correlated movements (represented by yellowish green) and regions with zero or uncorrelated movements (represented by yellow to deep yellow). This map helps in understanding the dynamic interactions within the binding site and the overall stability of the complex shown in Fig. 2H. It has been indicated in the graph that the binding-site residues where PREPi bonded showed a positive correlation, which might be due to the adopted interactions of this compound with active site residues.

The observed binding interactions and docking score of PREPi along with the stability and compactness of the complex align with the expected behavior for a well-bound ligand–enzyme complex (Fig. 2D). The binding interactions, minimal docking scores, RMSD, RMSF, and RoG results are consistent with previous studies and theoretical predictions. The findings indicate that the ligand has successfully bound to the enzyme and stabilized the structure, which could be critical for its biological function or potential therapeutic applications. While the simulation provides valuable insights, further studies with longer simulation times or additional systems may be necessary to fully validate these findings. Moreover, the 200 ns MD simulation results demonstrate that the ligand–enzyme complex remains stable, with a compact and rigid structure throughout the simulation. The RMSD, RMSF, and RoG analyses confirm the stability and effective binding of the ligand. The DCCM offers further insights into residue interactions within the binding pocket. These results provide a solid foundation for understanding the dynamics and energetics of the ligand–enzyme complex. The structure–activity relationship (SAR) of PREPi reveals that its potent inhibitory activity (IC_50_ = 20.05 ± 1.6 μM) stems from synergistic interactions in PREP's active site. The hydroxyl group at C-1 forms a crucial hydrogen bond with Arg_252_ (Fig. 2D), while the extended aliphatic chain (C2–C25) mediates extensive hydrophobic contacts with Phe_476_ and occupies a deep binding pocket. This dual functionality, polar anchoring through the hydroxyl group combined with hydrophobic stabilization *via* the pentacosyl tail, enables PREPi to competitively inhibit PREP more effectively than smaller phenolic analogs. Molecular dynamics simulations confirm that the flexible aliphatic chain adjusts to the active site contours, sustaining stable binding interactions, while the cyclohexanol core provides optimal steric hindrance. Taken together, these features explain PREPi's > 7-fold greater potency compared to syringic acid (IC_50_ = 155.13 μM) and other isolated compounds, which lack either the optimal hydrogen bonding geometry or comparable hydrophobic interactions.

### 3.5. Effect of PREPi on PA-treated HepG2 cells

The results of the cell viability assay demonstrate a dose-dependent increase in cell viability and a protective effect of the PREPi compound against PA-induced toxicity in HepG2 cells. HepG2 cells treated with PREPi or Kyp-2047, without exposure to palmitic acid (PA), showed improved cell morphology and exhibited no cytotoxicity, as compared to the standard inhibitor (Kyp-2047), as shown in Fig. 2I. Treatment of HepG2 cells with 500 μM PA alone led to a significant reduction in cell viability when compared to the control group, confirming the cytotoxic effect of PA at this concentration. In Fig. 2J, the effect of PREPi in co-treatment (PREPi + PA) is illustrated, showing a marked increase in OD values compared to PA treatment alone. This suggests that PREPi significantly rescues cells from PA-induced toxicity. PREPi exhibited the greater lipotoxicity amelioration at 15 μM, with reduced efficacy at higher (20–25 μM) and lower doses (5–10 μM). The decline at suboptimal doses (<15 μM) may imply insufficient target engagement, while the decline at elevated doses may reflect alternative inhibitory mechanisms. Thus, 15 μM represents the optimal balance between potency and solubility under the tested conditions. Based on these findings, we selected 15 μM as the optimal dose for subsequent experiments.

This cytotoxicity is consistent with established mechanisms of lipotoxicity in hepatocytes, where high concentrations of saturated fatty acids such as PA induce endoplasmic reticulum (ER) stress, oxidative stress, and mitochondrial dysfunction, ultimately leading to apoptosis.^45,46^ Based on these findings, a concentration of 500 μM PA was selected as the optimal dose for subsequent co-treatment experiments, as it provides a balance between inducing significant apoptosis and maintaining measurable cell viability, thereby avoiding complete cell death. Fig. 2K shows that TG levels in HepG2 PA co-treated with 15 μM PREPi are lower than in cells treated with PA alone. PA significantly increased TG levels, indicating lipotoxicity, while PREPi co-treatment reduced TG levels, suggesting a protective effect against PA-induced lipid accumulation. This implies that PREPi may mitigate lipotoxicity by interfering with lipid metabolism pathways. It also implies that PREPi mitigate PA-induced apoptosis, due to its PREP inhibitory activity. PREP potentially participates in many pathological processes, including oxidative stress, inflammation, and autophagy. As reported earlier, PREP is implicated in oxidative stress and related MAFLD and NASH symptoms, including lipid overload. PREP inhibition has been shown to reduce the production of ROS and could provide a potential approach to achieve hepatocyte protection.^28,47,48^ Collectively, the results demonstrate that 15 μM PREPi effectively protects HepG2 cells from PA-induced cytotoxicity.

**Fig. 3:**
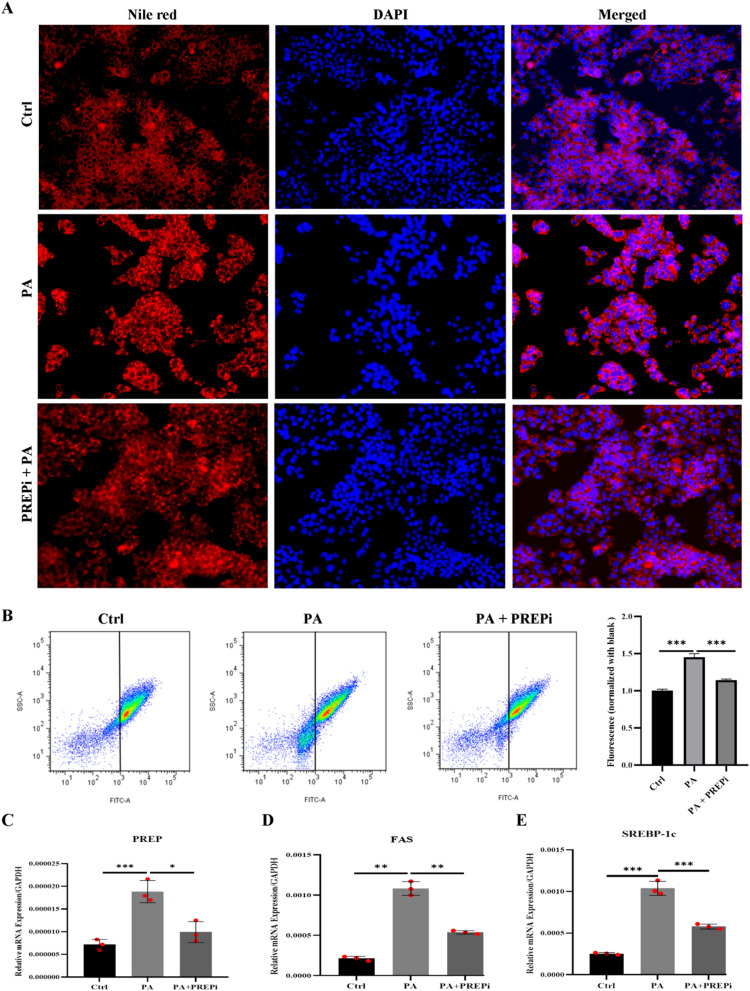
Effects of PREPi on lipid droplet accumulation in Palmitic Acid (PA)-Treated HepG2 cell. (A) Nile red staining images in control and treatment groups. (B) Lipid quantification of Nile red-stained cells *via* fluorescence-activated cell sorting (FACS). (C) Relative mRNA expression analysis of PREP (prolyl endopeptidase) in control, PA alone, and in combination with PREPi. (D) Relative mRNA expression analysis of FAS (fatty acid synthase) in all three groups. (E) Relative mRNA expression analysis of SREBP-1c (Sterol Regulatory Element-Binding Protein-1c) expression in all three groups. All data are presented as means ± SD. Statistical significance is indicated as follows: *p* < 0.05 (*), *p* < 0.01 (**), *p* < 0.001 (***), *p* < 0.0001 (****), “ns” denotes non-significant difference.

The Nile red staining assay revealed a significant increase in lipid droplet accumulation in HepG2 cells following PA exposure, indicating PA-induced lipotoxicity (Fig. 3A). In contrast, co-treatment with PREPi effectively reduced lipid droplet formation, suggesting that PREPi ameliorates PA-induced lipid accumulation. This reduction in lipid deposition implies that PREPi modulates lipid metabolism, enhances lipid clearance, or suppresses lipogenic pathways, thereby mitigating hepatocellular lipid overload. These findings align with the hypothesis that PREPi may serve as a therapeutic agent against lipotoxicity-induced metabolic stress, a key hallmark of MAFLD and NASH.^26^ Further validation of PREPi's lipotoxicity-reducing effects was obtained through flow cytometry-based lipid quantification (Fig. 3B), which showed a substantial increase in lipid fluorescence intensity in PA-treated cells, confirming excess lipid accumulation. Notably, PREPi co-treatment significantly lowered lipid fluorescence, reinforcing its role in counteracting PA-induced lipid overload and preserving cellular homeostasis. PA-induced lipotoxicity not only disrupts lipid homeostasis but also triggers ER stress and mitochondrial dysfunction in hepatocytes, leading to cell dysfunction.^11,49^ However, hepatocytes possess intrinsic mechanisms for recovery, including the activation of lipid catabolic pathways and antioxidant defence systems, which can be enhanced by therapeutic interventions such as PREPi.^26,28,50^

**Fig. 4:**
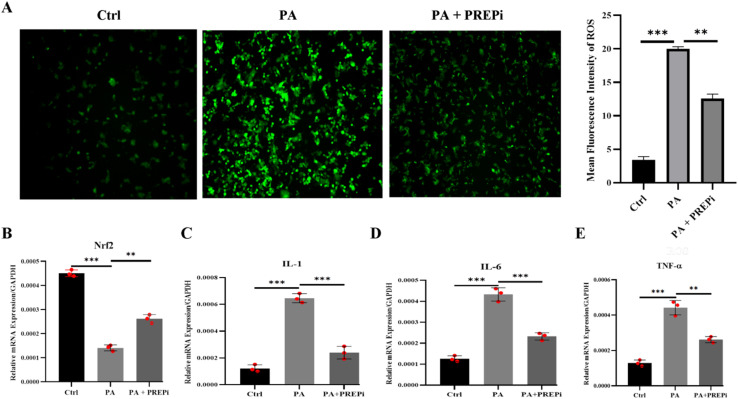
Reactive Oxygen Species (ROS) levels and gene expression analysis in PA-treated HepG2 cells with and without PREPi treatment. (A) Analysis of ROS levels in all three treatment groups of HepG2 cells using 2′,7′-dichlorodihydrofluorescein diacetate (DCFH-DA). (B) Mean relative mRNA expression analysis of Nrf2 (Nuclear factor erythroid 2-related factor 2) using RT-qPCR (C) mean relative mRNA expression analysis of IL-1 (Interleukin-1) using RT-qPCR. (D) Mean relative mRNA expression analysis of IL-6 (Interleukin-6) using RT-qPCR. (E) Mean relative mRNA expression analysis of TNF-α (Tumor Necrosis Factor-alpha) using RT-qPCR. All data are presented as means ± SD. Statistical significance is indicated as follows: *p* < 0.05 (*), *p* < 0.01 (**), *p* < 0.001 (***), *p* < 0.0001 (****), “ns” denotes non-significant differences.

In addition to lipid accumulation, PA exposure led to a significant increase in ROS levels (Fig. 3C), reflecting oxidative stress and cellular damage. Elevated ROS production is a key contributor to hepatic inflammation and mitochondrial dysfunction in MAFLD progression. Hepatocytes can recover by combating oxidative stress, as well as through mitochondrial quality control mechanisms.^51^ Importantly, PREPi co-treatment markedly reduced ROS levels, suggesting that PREPi exerts potent antioxidant properties, mitigating PA-induced oxidative stress and protecting hepatocytes from oxidative damage. As expected, PA induces the expression of PREP (Fig. 3C) in HEPG2 cells, which subsequently leads to a reduction in the expression of Nrf2 antioxidant genes. This reduction in Nrf2 expression likely contributes to an increase in ROS levels, as Nrf2 plays a critical role in the cellular defence against oxidative stress. In contrast, inhibition of PREP results in the upregulation of Nrf2 (Fig. 4B), enhancing the antioxidant response and reducing ROS levels, as evidenced by qPCR analysis. The ROS-combating effect of PREPi further supports its potential role in attenuating lipotoxicity-associated oxidative stress and preserving hepatocyte integrity. Together, these results highlight the dual protective role of PREPi in reducing lipid accumulation and oxidative stress, reinforcing its therapeutic potential in NASH and other metabolic liver diseases characterized by lipid dysregulation and oxidative injury. Several novel and well-established compounds have been investigated for their protective effects against lipotoxicity and oxidative stress in hepatocytes. Resveratrol, a polyphenolic compound, has been shown to mitigate PA-induced lipotoxicity by enhancing mitochondrial function and activating the SIRT1/AMPK pathway, thereby reducing lipid accumulation and oxidative damage.^52^ Additionally, berberine, an alkaloid, has demonstrated hepatoprotective effects by modulating lipid metabolism and reducing ROS production through the activation of the Nrf2/ARE signalling pathway.^53,54^ Furthermore, curcumin is shown to alleviate hepatocyte lipotoxicity by inhibiting endoplasmic reticulum stress and suppressing pro-inflammatory cytokine release, thereby maintaining cellular homeostasis.^55^ Studies have shown that targeting PREP can ameliorate oxidative stress status in PA-exposed HepG2 cells and fatty liver in mice, which may be due to the activation of the Nrf2/HO-1 pathway axis.^26^ These results indicate that a specific inhibitor of PREP may offer a novel therapeutic target for the prevention and treatment of NAFLD/NASH.^31^ Our findings, combined with existing literature,^26^ suggest PREP inhibition may enhance Nrf2-mediated antioxidant defences through multiple potential pathways. First, PREP could directly process Nrf2 or its negative regulator Keap1. Alternatively, PREP might modulate upstream signalling cascades (*e.g.*, p62/SQSTM1 accumulation) that activate Nrf2. The role of specific master metabolic pathways, like the AMPK/mTORC1 pathway, in the lipogenic modulation of hepatocyte steatosis and its interaction with inflammation and Nrf2/ARE signalling pathways can further unlock the PREPi mechanism of action.^56,57^

To evaluate the effects of PREPi on gene expression in PA-treated HepG2 cells, the qPCR analysis was performed on key lipogenic and inflammatory markers, including PREP, FAS, SREBP-1c, IL-1, IL-6, and TNF-α.^58,59^ As expected, PA treatment significantly upregulated PREP expression, supporting its established role in steatohepatitis pathogenesis. Notably, co-treatment with PREPi significantly reduced PREP expression, reinforcing its potential as a PREP inhibitor. Our finding conforms to the previously reported role of PREP overexpression in lipotoxicity and inflammation in MAFLD.^26,47^ The significant upregulation of PREP expression in palmitic acid (PA)-treated HepG2 cells is consistent with previous studies that associate PREP with lipid metabolism dysregulation and inflammation in the liver.^26,28^ In contrast, co-treatment with PREP inhibitor (PREPi) resulted in a reduction in PREP expression, although this change was not statistically significant. This suggests that PREPi likely inhibits the functional activity of PREP rather than directly impacting its gene expression or translation. Therefore, the observed decrease in PREP activity in the co-treatment group can be attributed to the inhibition of PREP's functional activity, rather than any alterations in its transcriptional or translational regulation (Fig. 3C). Consequently, the marked increase in lipogenic markers FAS and SREBP-1c in PA-treated cells reflects enhanced lipid accumulation and dysregulated lipid metabolism, which was effectively rescued with PREPi treatment, supporting the idea that PREP inhibition ameliorates steatohepatitis hallmarks. PREPi treatment significantly downregulated both FAS and SREBP-1c, suggesting its role in reducing hepatic lipogenesis and lipid storage (Fig. 3D and E). The pro-inflammatory cytokines IL-1, IL-6, and TNF-α were also significantly upregulated in PA-treated cells, indicating a robust inflammatory response. Furthermore, PREPi administration effectively suppressed the expression of all three inflammatory markers, demonstrating its potent anti-inflammatory effects in the PA-induced MAFLD model. This significant upregulation of pro-inflammatory cytokines IL-1, IL-6, and TNF-α in PA treatment is indicative of a robust inflammatory response and pathophysiology of MAFLD.^60^ The suppression of these inflammatory markers upon PREPi treatment reinforces the potential of PREP inhibition in modulating inflammation and protecting hepatocytes from PA-induced metabolic dysfunction (Fig. 4C and D). Overall, these mRNA expression findings strongly support the notion that PREPi attenuates steatohepatitis pathology by modulating PREP function, reducing lipogenesis, and suppressing inflammation, thereby protecting hepatocytes from PA-induced metabolic dysfunction. These results further reinforce the therapeutic potential of PREPi as a novel agent for managing liver diseases and steatohepatitis.

### 3.6. PREPi alleviates steatohepatitis *in vivo*

**Fig. 5:**
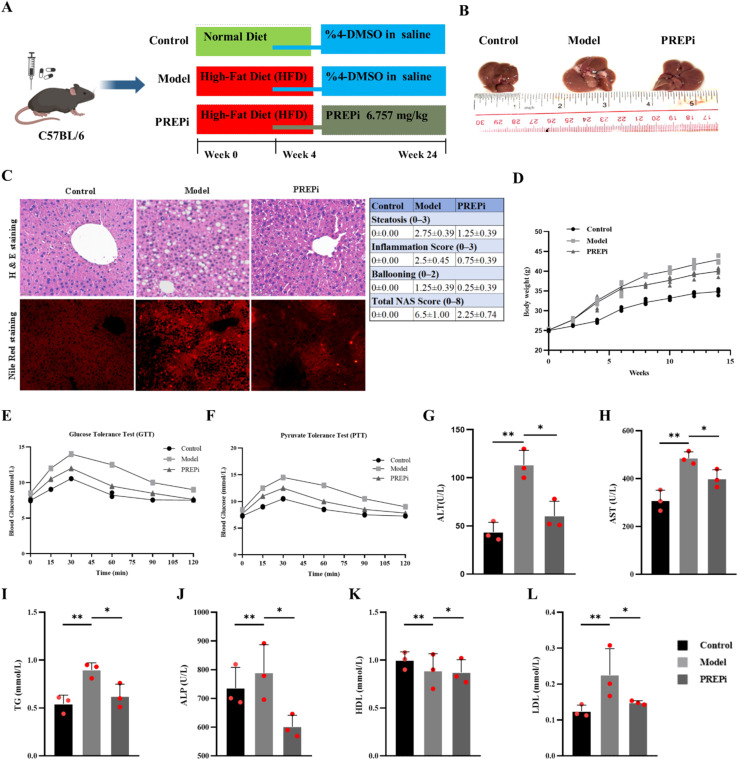
PREPi alleviates steatohepatitis in a High-Fat Diet (HFD) mouse model. (A) Experimental design and treatment timeline for establishing the steatohepatitis model in C57BL/6 mice. (B) Representative liver images from control, model (HFD-fed), and PREPi-treated groups. (C) Hematoxylin and Eosin (H & E) staining of liver tissues along with quantification of liver histology using the NAFLD Activity Score (NAS) in control, model, and PREPi-treated groups. (D) Mice body weight progression curve across all three treatment groups over 24 weeks. (E) Glucose Tolerance Test (GTT) results in control, model, and PREPi-treated groups. (F) Pyruvate Tolerance Test (PTT) results in control, model, and PREPi-treated groups. (G–H) ELISA-based analysis of serum liver enzyme levels (ALT and AST) in control, model, and PREPi-treated groups. (I–L) Lipid profile analysis, including Total Cholesterol (TC), Alkaline Phosphatase (ALP), Low-Density Lipoprotein (LDL), and High-Density Lipoprotein (HDL) in control, model, and PREPi-treated groups. All data are presented as means ± SD. Statistical significance is indicated as follows: *p* < 0.05 (*), *p* < 0.01 (**), *p* < 0.001 (***), *p* < 0.0001 (****), “ns” denotes non-significant differences.

The steatohepatitis model was successfully established by a high-fat diet as Fig. 5A shown. In comparison to the control group, mice fed a high-fat diet (HFD) for 24 weeks exhibited an increase in liver size and body weight (Fig. 5B and D).^61^ However, when mice were subsequently administered with PREPi (intravenously) from week four onwards, a significant decrease in body weight and liver size was observed when compared to the model group (*P* < 0.05). The results showed that PREPi could inhibit the weight gain of HFD mice significantly. To further prove that PREPi can significantly ameliorate steatohepatitis, the liver index and body fat index of the mice were evaluated as shown in Fig. 5C–L.

In the control group, the liver appeared normal in size, with a regular shape, smooth surface, and healthy ruddy colour, showing no signs of oedema. In contrast, the liver of HFD-fed mice exhibited slight enlargement, whitened edges, and a fragile texture. Notably, the liver morphology of PREPi-treated mice showed significant improvement, with minimal hypertrophy and whitening at the edges compared to the HFD group (Fig. 5B). Quantitative histological analysis of liver tissues (H & E stained) revealed that the hepatic lobules in the control group maintained a well-defined and intact structure, with hepatic cords arranged radially around the central vein. The hepatic sinusoids were visible, and hepatocytes were uniform in size. While HFD-fed mice exhibited significant steatosis, lobular inflammation, and ballooning, resulting in a total NAS of 6.5 ± 0.5. Furthermore, in the HFD group, hepatocytes exhibited increased volume, cytoplasmic fat vacuoles of varying sizes, displaced nuclei, and compressed, narrowed sinusoids. In contrast, PREPi-treated mice had markedly lower NAS (2.25 ± 0.48, *p* < 0.01), indicating improved liver architecture. In comparison, the liver tissue of PREPi-treated mice also displayed signs of fatty degeneration; however, the number of degenerated cells and cytoplasmic lipid droplets was markedly reduced. Control mice had no detectable pathology (0.0 ± 0.0). These results confirm that PREPi reduces key pathological features of NASH (Fig. 5C). These results are aligned with the previous studies about the role of PREP inhibition in the HFD-fed mice models.^26,28^ Overall, our results provide compelling evidence that PREPi attenuates HFD-induced lipid accumulation in the liver. Notably, other PREP inhibitors, *e.g.*, (Kyp-2047) have shown favorable toxicity profiles in chronic models,^62^ suggesting PREPi may share similar properties pending further studies.

To evaluate the metabolic and hepatoprotective effects of PREPi, we assessed glucose homeostasis (Glucose Tolerance Test (GTT) and Pyruvate Tolerance Test (PTT)) and liver function markers in the control, HFD model, and HFD + PREPi treatment groups. Mice in the HFD group exhibited significant impairments in glucose metabolism, as indicated by elevated fasting glucose levels, increased peak glucose concentrations, and delayed glucose clearance compared to the control group. These results are typical of steatohepatitis in mice.^28,61^ In the PTT, the HFD group showed higher baseline glucose levels (8.4 mM L^−1^, *p* < 0.05), increased peak glucose (14.5 mM L^−1^, *p* < 0.01), and delayed glucose clearance (9.0 mM L^−1^ at 120 min, *p* < 0.05) compared to the control group (baseline: 7.3 mM L^−1^, peak: 10.5 mM L^−1^). This suggests dysregulated gluconeogenesis and impaired hepatic insulin sensitivity in the HFD group (Fig. 5F). Notably, PREPi administration significantly improved these parameters, as evidenced by lower fasting glucose (7.8 mM L^−1^, *p* < 0.05 *vs.* HFD), reduced peak glucose (12.5 mM L^−1^, *p* < 0.05), and enhanced glucose clearance (7.8 mM L^−1^ at 120 min, *p* < 0.05). Similarly, in the GTT, the HFD group exhibited marked glucose intolerance, with significantly higher baseline glucose (8.5 mM L^−1^, *p* < 0.05), peak glucose (14.0 mM L^−1^, *p* < 0.01), and delayed glucose clearance (9.0 mM L^−1^ at 120 min, *p* < 0.05) compared to the ND group (baseline: 7.5 mM L^−1^, peak: 10.5 mM L^−1^). PREPi treatment significantly improved glucose tolerance, as shown by lower peak glucose (12.0 mM L^−1^, *p* < 0.05) and faster clearance (7.5 mM L^−1^ at 120 min, *p* < 0.05), suggesting that PREPi enhances insulin sensitivity and hepatic glucose regulation (Fig. 5E).

HFD-induced hepatic injury and dyslipidemia were evident in the model group, characterized by marked increases in serum ALT (100–130 U L^−1^, *p* < 0.01), AST (365–515 U L^−1^, *p* < 0.01), and ALP (696–892 U L^−1^, *p* < 0.01). These elevated levels indicate hepatic inflammation and dysfunction, key features of steatohepatitis pathology.^61^ Additionally, the HFD group displayed dyslipidemia, with significantly higher total cholesterol (CHO: 3.13–3.77 mmol L^−1^, *p* < 0.01) and triglycerides (TG: 0.81–0.95 mmol L^−1^, *p* < 0.05) compared to the control group. Intravenous (IV) administration of PREPi significantly alleviated these pathological changes, as demonstrated by lower ALT (51–78 U L^−1^, *p* < 0.05), AST (394–479 U L^−1^, *p* < 0.05), and ALP levels, indicating improved hepatic function. Moreover, PREPi treatment effectively reduced dyslipidemia, with a significant decrease in TG levels (0.51–0.76 mmol L^−1^, *p* < 0.05) and total cholesterol (2.05–2.47 mmol L^−1^, *p* < 0.05). The improvement in lipid metabolism and liver function aligns with PREPi's proposed mechanism of action, which involves decreasing hepatic inflammation and reducing fats.^26,28^

## 4 Conclusion

In this study, we isolated and characterized the main bioactive compound, (±)-2-pentacosylcyclohexanol (PREPi), responsible for the hepatoprotective effects of *M. germanica*. Mechanistically, PREPi significantly inhibited PREP activity, as demonstrated through *in vitro* enzymatic assays, molecular docking, and molecular dynamics simulations, which confirmed its stable binding at the enzyme's active site. Although PREPi showed no signs of acute toxicity in our model, its long-term safety requires further validation to rule out adverse effects. Until such data are available, clinical translation should proceed cautiously. Functionally, PREPi mitigated PA-induced lipotoxicity, oxidative stress, and lipid accumulation in HepG2 cells, evidenced by reduced intracellular triglycerides, diminished ROS generation, and improved cell viability. In the HFD-induced steatohepatitis mouse model, IV administration of PREPi significantly attenuated hepatic pathology, as reflected by improved liver histopathology, reduced serum ALT and AST levels, and enhanced glucose and lipid metabolism. Given the limitations of current pharmacological interventions, PREPi's ability to target oxidative stress, particularly mitochondrial dysfunction in HFD-induced steatohepatitis makes it a strong candidate for further preclinical and clinical development. Future studies should prioritize elucidating the precise molecular link between PREP inhibition and Nrf2 activation, including potential effects on Keap1 degradation or kinase signaling pathways. These types of mechanistic investigations of PREP using PREPi are plausible, as it is naturally occurring and non-cytotoxic. This study highlights the therapeutic potential of a natural PREP inhibitor, offering promising avenues for the development of novel treatment strategies and providing the scientific basis for the pharmacological use of this medicinal plant in the context of fatty liver disease.

## Author contribution

Khair Ullah contributed to conceptualization, investigation, drafting, and visualization. Tasneef Azam worked on conceptualization, investigation, and visualization. Khairullah and Abdul Sammad carried ou the drafting and wrote the original manuscript. Tanveer Ahmed performed data curation and formal analysis. While Abdul Wadood focused on methodology and visualization. Ghulam M. Mushraf assisted with visualization while Muhammad Fawad Ali helped in the final revision. Yinxiong Li and Bina Shaheen Siddiqui supervised and edited the manuscript. All authors reviewed and approved the final version.

## Conflicts of interest

The authors affirm that this research was conducted without any commercial or financial affiliations that could be interpreted as a potential conflict of interest.

## Supplementary Material

RA-015-D5RA03146J-s001

## Data Availability

Data supporting this study (novel prolyl endopeptidase inhibitor from *M. germinica* alleviates metabolic dysfunction-associated fatty liver disease) are available from the corresponding author upon reasonable request. Supplementary information is available. See DOI: https://doi.org/10.1039/d5ra03146j.
