# Supplementary figures and images for: Novel prolyl endopeptidase inhibitor from Myricaria germanica alleviates steatohepatitis
Source: RSC Adv. 2025 Oct 7;15(44):37245–62. doi: 10.1039/d5ra03146j (PMC12501607; doi:10.1039/d5ra03146j)

### Prolyl endopeptidase inhibitory activity-guided isolation of PREP inhibitor (PREPi)

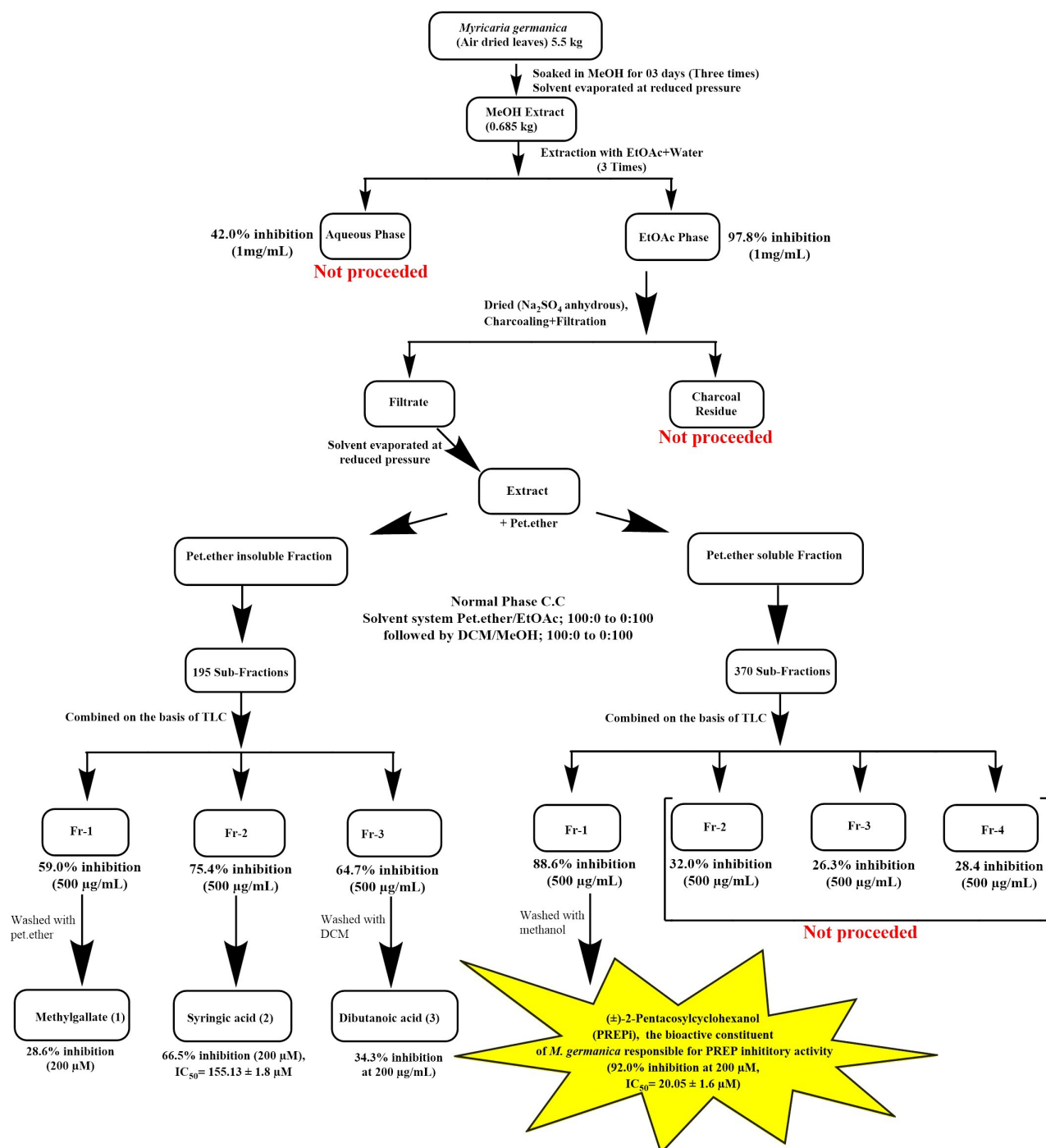

Supplement: RA-015-D5RA03146J-s001 [file RA-015-D5RA03146J-s001.pdf]
